# Classification of Gan Dan Shi Re Pattern and Gan Shen Yin Xu Pattern in Patients with Hepatitis B Cirrhosis Using Metabonomics

**DOI:** 10.1155/2018/2697468

**Published:** 2018-11-21

**Authors:** Chao-qun Zhao, Long Chen, Hong Cai, Wei-li Yao, Qun Zhou, Hui-ming Zhu, Yue Gao, Ping Liu, Xiao-jun Gou, Hua Zhang

**Affiliations:** ^1^Institute of Liver Disease, Shuguang Hospital, Shanghai University of Traditional Chinese Medicine, Key Laboratory of Liver and Kidney Diseases of Ministry of Education, Key Laboratory of Clinical Chinese Medicine, 258 Zhangheng Road, Pudong District, Shanghai 201203, China; ^2^Xiamen Hospital of Traditional Chinese Medicine, Fujian University of Traditional Chinese Medicine, Fujian 361000, China; ^3^E-Institute of Traditional Chinese Internal Medicine, Shanghai Municipal Education Commission, Shanghai University of Traditional Chinese Medicine, 1200 Cailun Road, Shanghai 201203, China; ^4^Central Laboratory, Baoshan District Hospital of Integrated Traditional Chinese and Western Medicine of Shanghai, Shanghai 201999, China

## Abstract

**Objective:**

This study aimed to analyze the differential metabolites and their metabolic pathways from the serum of patients with hepatitis B cirrhosis, with two typical patterns of Gan Dan Shi Re (GDSR) and Gan Shen Yin Xu (GSYX) based on the theory of traditional Chinese medicine (TCM). It also investigated the variation in the internal material basis for the two types of patterns and provided an objective basis for classifying TCM patterns using metabolomic techniques.

**Methods:**

The serum samples taken from 111 qualified patients (40 GDSR cases, 41 GSYX cases, and 30 Latent Pattern (LP) cases with no obvious pattern characters) and 60 healthy volunteers were tested to identify the differential substances relevant to hepatitis B cirrhosis and the two typical TCM patterns under the gas chromatography–time-of-flight mass spectrometry platform. The relevant metabolic pathways of differential substances were analyzed using multidimensional statistical analysis.

**Results:**

After excluding the influence of LP groups, six common substances were found in GDSR and GSYX patterns, which were mainly involved in the metabolic pathways of glycine, serine, threonine, and phenylalanine. Eight specific metabolites involved in the metabolic pathways of linoleic, glycine, threonine, and serine existed in the two patterns.

**Conclusions:**

The data points on the metabolic spectrum were found to be well distributed among the differential substances between the two typical TCM patterns of patients with hepatitis B cirrhosis using metabolomic techniques. The differential expression of these substances between GDSR and GSYX patterns provided an important objective basis for the scientific nature of TCM pattern classification at the metabolic level.

## 1. Introduction

Hepatitis B cirrhosis is one of the most fatal, refractory, and progressive liver diseases worldwide according to recent qualified epidemiological studies [1]. Based on its holistic and individualized diagnosis and treatment characteristics, traditional Chinese medicine (TCM) has unique advantages in treating hepatitis B cirrhosis by improving the clinical symptoms and liver function, reversing liver fibrosis, or even preventing the progression of cirrhosis.

The pattern “Zheng,” according to the transliteration of Chinese, is the core concept of TCM diagnosis, treatment, and determination of curative effect. Accurate pattern discernment is the core link to improve the clinical efficacy of TCM. Traditionally the pattern differentiation is based mainly on practitioners' own experience such as looking, smelling, talking, and feeling the pulse. Limited objective evidence also restricts the development of the study about the Chinese pattern. With the advancement of science and technology, many researchers tried to explain the pattern classification rules by modern scientific methods to make up for the deficiency of objectivity and reproducibility in pattern differentiation.

Metabolomics examines mainly the dynamic changes in the quality and quantity of metabolites produced in biological systems in response to pathophysiological reactions or genetic modification, thus finding the relative relationship between metabolites and pathophysiological changes in organisms [2]. It is consistent with the systematic and holistic view of TCM [3]. Studying the exogenous molecular compounds to understand the changing laws of the occurrence and development of the disease is of great significance in discussing the pathogenesis, diagnosis, treatment, and evaluation of the disease. It also provides methodological support for the objective and accurate diagnosis of patterns in TCM. As an effective method to study the physiological and pathological changes in body fluids and tissues, metabolomics was widely applied to TCM* in vitro* or* in vivo*, such as efficacy in evaluating Chinese medicine and its biochemical mechanism of action [4, 5], toxicity evaluation and toxicological biomarker identification of natural products [6], active fraction identification of prescription [7], and research on tongue coating [8].

Currently, finding blood and urine biomarkers for the diagnosis, treatment, and prognosis of liver cirrhosis is a hot spot in the liver disease research [9]. Guo et al. [10] used GC/MS methods to study the urine of healthy people and patients with hepatitis B cirrhosis. They found that the metabolic spectrum could be clearly separated between the two groups, providing the basis for diagnosing hepatitis B cirrhosis from the perspective of metabonomics. Yang et al. [11] analyzed serum metabolic profiles in healthy controls and patients with cirrhotic ascites and found potential biomarkers for diagnosing and treating liver fibrosis and cirrhosis. Tang et al. [12] compared different metabolites in serum and urine of patients with primary biliary cirrhosis (PBC) and healthy people using ultraperformance liquid chromatography coupled with quadrupole-time-of-flight mass spectrometry (UPLC-QTOF/MS). The results showed that the bile acid level increased and the carnitine content decreased with the development of PBC, suggesting that the two substances may be the potential biomarkers of PBC. These studies have indicated the important role of metabonomics technology in diagnosing liver cirrhosis. Mcphail et al. [13] examined 80 patients with decompensated cirrhosis, including 62 who survived and 18 who died, using plasma metabonomics analysis. The disorder of phosphatidylcholine and amino acid metabolism is related to the increase in mortality and the severity of the disease, providing the objective basis for accurately predicting the mortality of patients with decompensated cirrhosis. Based on GC/MS and LC/MS metabolomic techniques, the presence of specific compounds was preliminarily confirmed in the urine of patients with hepatitis B cirrhosis that could reflect the patterns of Gan Dan Shi Re (GDSR) and Gan Shen Yin Xu (GSYX) [14]. The metabolomic study on the dampness-heat pattern of chronic hepatitis B (CHB), nonalcoholic fatty liver disease (NAFLD), and chronic glomerulonephritis (CG) revealed five common biomarkers between the three diseases, including inosine, uridine, aspartic acid, oleic acid, and lactate, and their specific substances (27 substances in CHB, 28 in NAFLD, and 24 in CG) [15].

Based on previous findings, this study examined the typical GDSR and GSYX patterns of hepatitis B cirrhosis using healthy persons as the control group. Also, the study included an LP group (which means that the patients had no obvious clinical manifestation of the patterns or had little information, making it difficult to distinguish these patterns from others) as the control. The study was performed on typical GDSR and GSYX patterns of hepatitis B cirrhosis, a disease that could be treated using TCM. Qualitative and quantitative analyses of the metabolites (serum samples) were performed on the basis of gas chromatography–time-of-flight mass spectrometry (GC-TOF/MS) multiple combination techniques to determine the characteristic compound spectrum of hepatitis B cirrhosis and the two typical patterns of GSYX and GSYX. The present study aimed to provide data support for classifying and identifying the TCM pattern of refractory hepatitis B cirrhosis.

## 2. Materials and Methods

### 2.1. Study Participants

All the 111 patients in this study on hepatitis B cirrhosis were aged 18–65 years and admitted at Shuguang Hospital Affiliated to Shanghai University of TCM, Xiamen TCM Hospital, and Shanghai Putuo District Central Hospital between March 2016 and March 2017. A total of 60 healthy sex- and age-matched volunteers were selected from the Shuguang Hospital Health Examination Center. All the participants in the study signed informed consent.

#### 2.1.1. Inclusion Criteria

Participants were selected according to the standards of the Ishak Liver Fibrosis Grading Criteria and the Guidelines for the Prevention and Treatment of Chronic Hepatitis B (2015 Edition) [16], which was revised jointly by the Chinese Medical Association Hepatology Branch and the Infectious Diseases Branch, and in accordance with GDSR, GSYX [17], and LP [18] diagnostic criteria.

#### 2.1.2. Exclusion Criteria

The exclusion criteria were as follows: (1) age less than 18 years or more than 65 years; (2) a combination with other types of hepatitis such as hepatitis A, C, D, and E, or severe hepatitis; (3) a combination with heart, liver, hematopoietic, and neurological disorders, or drug allergy; (4) a combination with malignant tumors or connective tissue diseases; (5) pregnant or lactating women; (6) patients with anaphylaxis and other severe diseases; and (7) patients with mental disorders who could not cooperate with investigators.

### 2.2. Instruments and Reagents

High-purity methoxyamine hydrochloride, fatty acid methyl ester (C7–C30, FAME), anhydrous pyridine (99.5%), and anhydrous sodium sulfate were obtained from Sigma–Aldrich (MO, USA). Derivatization reagents MSTFA (containing 1% TMCS), methanol (Optima LC-MS), and* n*-hexane were purchased from Thermo Fisher (NJ, USA). Dichloromethane (99.5%), chloroform (99%), and acetone (99.5%) were purchased from China National Pharmaceutical Group Corporation (Beijing, China). Ultrapure water was prepared from a Millipore Reference ultrapure water system (MA, USA) equipped with an LC-MS filter. GC-TOF/MS (LECO Corp, MI, USA) based on silanization-derived GC-TOF/MS was used as an analytical platform for untargeted metabolomics.

### 2.3. Clinical Information Collection and Pattern Identification

Using the “Liver and Kidney Disease and Pattern Clinical Information Collection Form of the Key Laboratory of Ministry of Education,” the clinical information of the participants was collected, including basic information: gender, age, and ethnicity; physical examination: body temperature, heart rate, breathing, and blood pressure; biochemical examination: liver function, fibrosis index, and blood routine; and four examinations of TCM. Using the posthepatitis cirrhosis pattern rating scale, the clinical symptoms of TCM were quantified by five levels, with no symptoms rated as 0 points (1–4 points representing different degrees of severity) [19]. All information was input into the database. Three experts in the field gave the pattern differentiation using four examinations (including tongue photo) on the basis of inclusion criteria.

### 2.4. Collection of Blood Samples

BD Vacutainer vacuum blood collection tubes were used to collect 7 mL of whole blood from the fasting patients in the morning and kept undisturbed at 4°C for 2 h. The blood was separated and centrifuged (3000*g*, 15 min, 4°C) to obtain the serum. The serum was packed in tubes (400 *μ*L per tube) and stored at –80°C to avoid repeated freezing and thawing so that the sample remained stable.

### 2.5. GC-TOF/MS Detection

#### 2.5.1. Sample Pretreatment

Serum samples from the patients were prepared and stored for the detection. The detailed procedure of serum pretreatment is provided in the supplementary file (available here).

#### 2.5.2. Analysis Conditions

*(1) Chromatography*. Column Rxi-5MS (Restek Corporation, PA, USA), column parameters: 30 m (length) × 0.25 mm (internal diameter), 0.25 *μ*m (film thickness); oven temperature program 80°C (2 min), 80–300°C (12°C/min), 300°C (5.7 min); injection volume (*μ*L) 1; inlet temperature 270°C; injection mode: no split; carrier gas: helium (99.9999%); carrier gas flow rate (mL/min) 1.0; constant current; transmission line temperature: 270°C.

*(2) Ion Source Type*. EI, detection parameters: electron energy, –70 V; detector voltage, –1400 V; ion source temperature, 220°C; acquisition rate, 25 spectra/s; scan range, 50–500* m*/*z*.

### 2.6. Metabolic Pathway Analysis

The specific metabolites of different patterns by screening were introduced into the online system MetaboAnalyst for analyzing the metabolic pathways. It is generally believed that changes in key locations in the network have a serious impact on the occurrence of events. Therefore, the threshold was set to 0.10 [20] in this study, and the pathways above this threshold were classified as potential metabolic pathways. The metabolic pathways in this study were all generated using KEGG (http://www.genome.jp/kegg/).

### 2.7. Data Processing and Statistical Analysis

The raw data were automatically exported using the ChromaTOF software (v4.51.6.0, CA, USA) to the self-developed metabolomic macrometabolism software XploreMET (v2.0, Metabo-Profile, Shanghai, China) for baseline smoothing and correction, deconvolution, signal extraction from original chromatographic peaks and alignment, retention index correction, metabolite identification, data preprocessing (normalization and standardization), statistical analysis, metabolic network analysis, and reporting. The analysis was performed using SPSS 21.0 statistical software (IBM, NY, USA), in which the measurement data conforming to the normal distribution and the homogeneity of variance were described as mean ± standard deviation ($\overset{-}{x}\pm s$). The comparison between multisample groups was analyzed by variance for nonconformity with normal distribution or variance. The measurement data were described as the median M (Q1, Q3). The Wilcoxon rank-sum test was used for comparison between the two groups. The Kruskal–Wallis* H* test was used for the multisample comparison of the group design; the count data were described as the relative number (%). The groups were compared using the* χ*2 test. Setting *α* = 0.05, a* P* value less than 0.05 suggested that the difference was statistically significant.

## 3. Results

### 3.1. Demographic Characteristics

The study comprised 171 participants, including 30 patients having an LP pattern, 40 patients having a GDSR pattern, 41 patients having a GSYX pattern, and 60 healthy volunteers.  Table 1 shows their demographic characteristics.

### 3.2. Physical and Chemical Examination

No significant difference in alanine aminotransferase (ALT) was found between the groups (*P* > 0.05). The level of albumin (ALB) in the LP group; the levels of total bilirubin (TBil), Direct Bilirubin (DBil), aspartate transaminase (AST), alkaline phosphatase (ALP), gamma-glutamyl transpeptidase (GGT), total biliary acid (TBA), and ALB in the GDSR group; and the levels of TBil, ALP, GGT, TBA, and ALB in the GSYX group were statistically significantly different compared with the healthy group (*P* < 0.05). Significant differences in the levels of TBil, DBil, AST, ALP, TBA, and ALB in the GDSR group and the levels of TBA and ALB in the GSYX group were found compared with the LP group (*P* < 0.05). A statistically significant difference in the levels of TBil and DBil was observed between the GDSR and GSYX groups (*P* < 0.05) (Table 2).

### 3.3. Metabolites

#### 3.3.1. Multivariate Data Analysis

**Figure 1:**
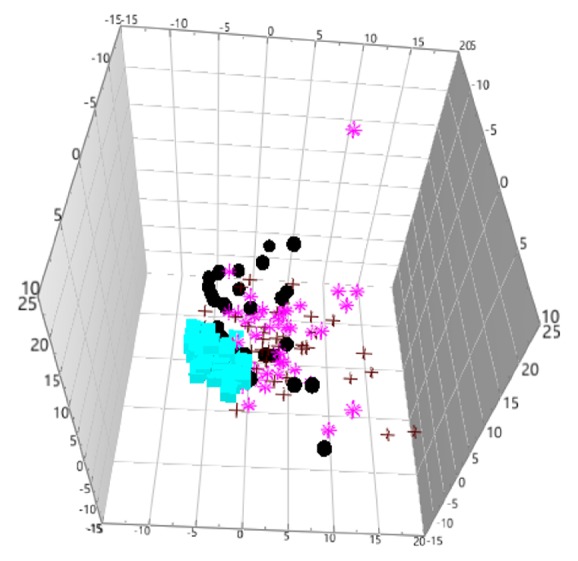
Unsupervised PCA analysis of the four groups (“black circle” LP, “red plus” GDSR, “pink asterisk” GSYX, “blue square” HG).

**Figure 2:**
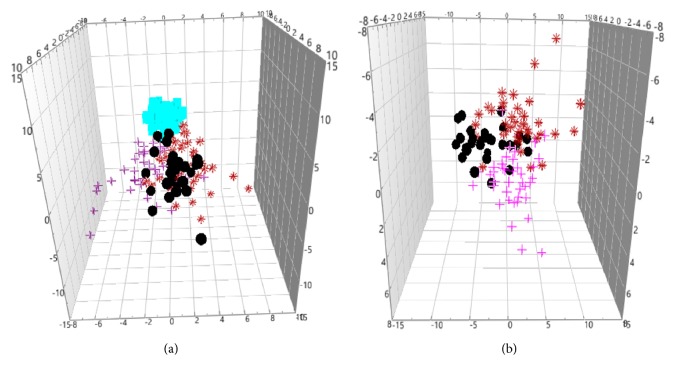
(a) PLS-DA score plot of disease and health groups. (b) PLS-DA score plot of disease groups (“black circle” LP, “red asterisk” GDSR, “pink plus” GSYX, “blue square” HG).

**Figure 3:**
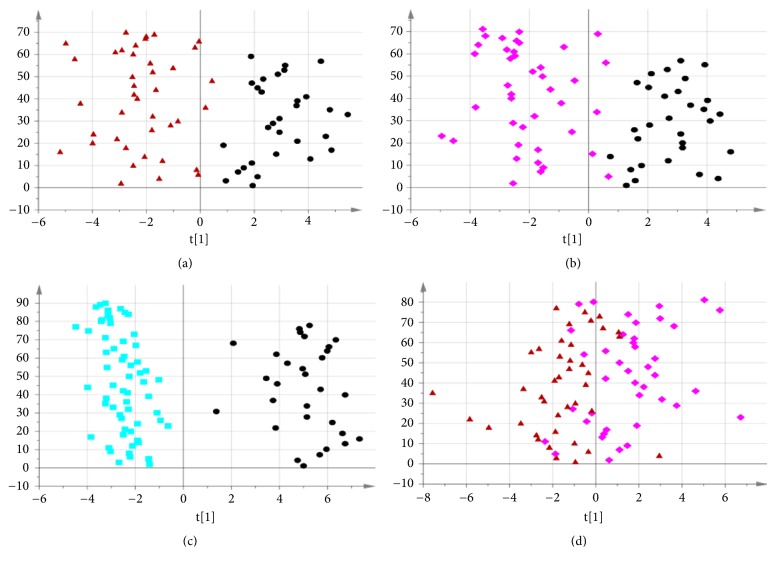
(a) OPLS-DA metabolic map of LP (the black circle) and GDSR (the red triangle). (b) OPLS-DA metabolic map of LP and GSYX (the pink diamond). (c) OPLS-DA metabolic map of LP and HG (the blue square). (d) OPLS-DA metabolic map of GDSR and GSYX.

The unsupervised principal components analysis (PCA) method was used to observe the natural distribution of each group. Separation trends were found in the LP, GDSR, and GSYX compared with HG using three-dimensional PCA analysis (see Figure 1). For further validation, partial least square discriminant analysis (PLS-DA) was used to verify a good separation between disease and healthy groups and among disease groups (see Figure 2). The orthogonal partial least square discriminant analysis (OPLS-DA) method was used for further analysis. The metabolic spectrum between the LP and GDSR, LP and GSYX, and LP and HG could be completely distinguished on the principal component t [1] (each point in the figure represents the information of the sample metabolite; the farther it was from the original point, the greater the contribution to distinguish the two groups is, leading to metabolites with larger differences). The metabolic spectrum of serum in GDSR and GSYX also showed a tendency of separation, suggesting differences in the endogenous metabolites between the two groups (Figure 3).

#### 3.3.2. Identification of Different Metabolites

The differential variables were screened with variable importance in projection (VIP) >1.5 (*P *< 0.05) based on the OPLS-DA analysis results for each group using endogenous metabolite database JIALIB and standard substances, aiming to look for metabolites highly related to liver cirrhosis. Compared with the healthy group, the results showed that 22 different metabolites were obtained in the LP group (Table 3). Compared with the LP group, 20 differential metabolites were obtained in the GDSR group (Table 4) and 18 in the GSYX group (Table 5).

#### 3.3.3. Analysis of Common Metabolites of Different Patterns

**Figure 4:**
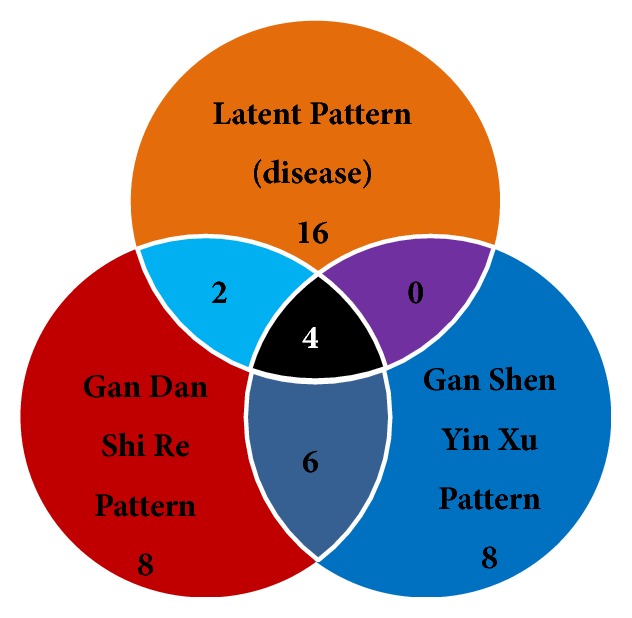
Venn diagram of differential substances in three patterns. With the removal of disease information (LP), eight specific metabolites were found in GDSR, eight specific metabolites were found in GSYX, and six common substances were found in two typical patterns.

The GDSR and GSYX groups were compared with the LP group to find out the specific metabolites representing only the two typical patterns. Eight specific metabolites, including citrulline, urea, myristic acid, palmitic acid, palmitoleic acid, linoleic acid, glycolic acid, and petroselinic acid, were found in GDSR. Also, eight specific metabolites, including L-threonine, pyroglutamic acid, L-arabitol, 1,5-anhydrosorbitol, glyceric acid, L-pipecolic acid, glutaric acid, and alpha-tocopherol were found in GSYX. Further, six common substances, including D-2-hydroxyglutaric acid, 2-hydroxybutyric acid, L-phenylalanine, L-arabinose, L-serine, and quinic acid were found in both the two patterns (Table 6 and Figure 4).

### 3.4. Metabolic Pathways

#### 3.4.1. GDSR

**Figure 5:**
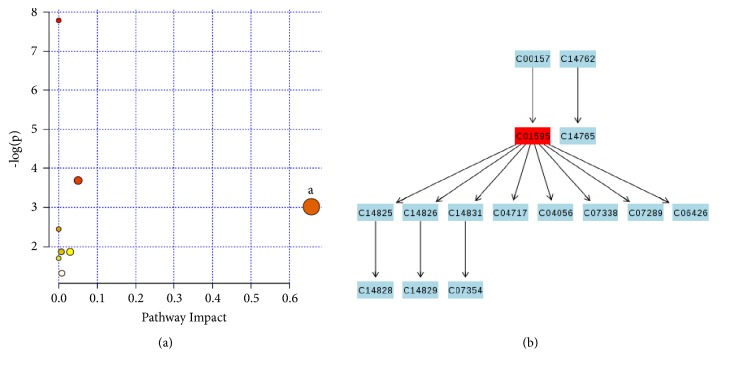
(a) Summary of pathway analysis. (b) Linoleic acid metabolism.

Eight screened substances, including citrulline, urea, myristic acid, palmitic acid, palmitoleic acid, linoleic acid, glycolic acid, and petroselinic acid of GDSR, were used for analyzing the metabolic pathways. The results showed one important metabolic pathway of linoleic acid metabolism. Metabolic pathways were generated using KEGG (http://www.genome.jp/kegg/) (Figure 5).

#### 3.4.2. GSYX

**Figure 6:**
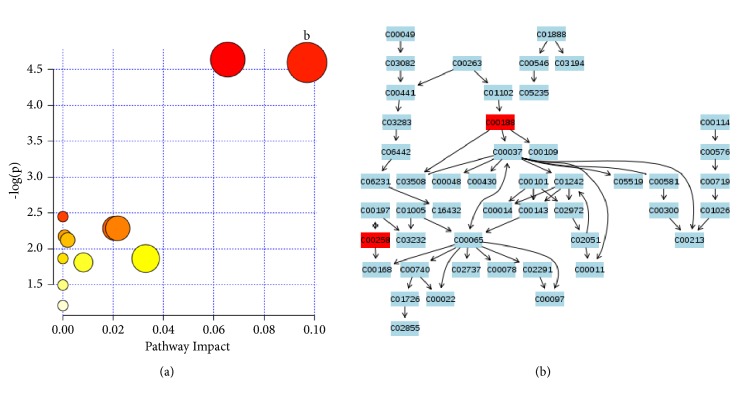
(a) Summary of pathway analysis. (b) Glycine, serine, and threonine metabolism.

The metabolic pathways of the screened eight substances, including L-threonine, pyroglutamic acid, L-arabitol, 1,5-anhydrosorbitol, glyceric acid, L-pipecolic acid, glutaric acid, and alpha-tocopherol in GSYX, were analyzed. An important metabolic pathway of glycine, serine, and threonine was found, as shown in Figure 6.

#### 3.4.3. Common Metabolic Pathways of GDSR and GSYX

**Figure 7:**
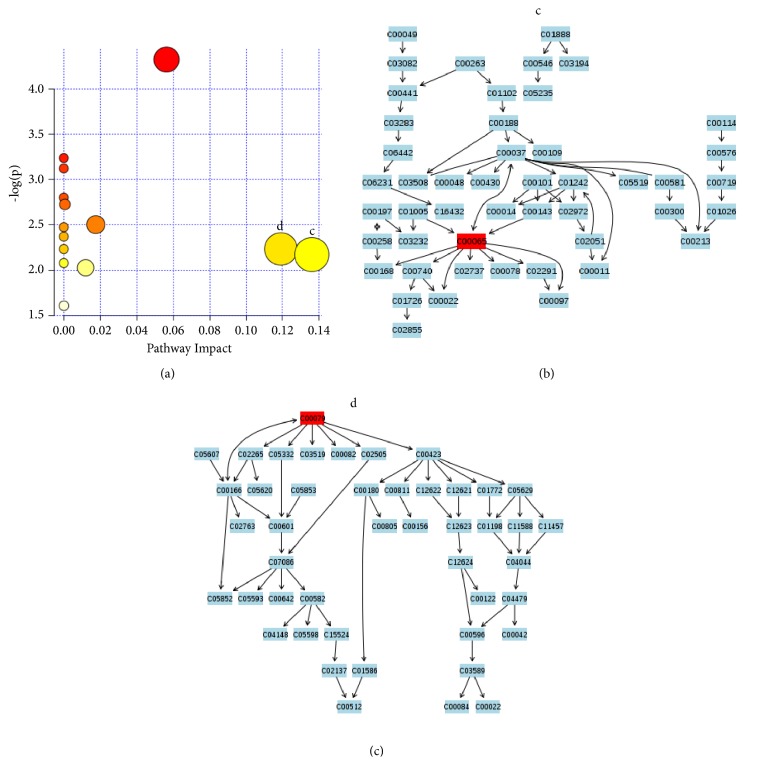
(a) Summary of pathway analysis. (b) Glycine, serine, and threonine metabolism. (c) Phenylalanine metabolism.

Six common metabolites in GDSR and GSYX, including D-2-hydroxyglutaric acid, 2-hydroxybutyric acid, L-phenylalanine, L-arabinose, L-serine, and quinic acid were used to analyze the metabolic pathways. Two important metabolic pathways of glycine, serine, and threonine and phenylalanine metabolism were found in two patterns (Figure 7).

## 4. Discussion

Metabolomics has been applied to explore the nature of TCM pattern in recent years. Gou et al. [21] used GC/MS metabolomics to analyze the urine of children with asthma having lung spleen qi deficiency, those having qi and yin deficiency pattern, and normal children and finally found differential markers that distinguished children's asthma. Cheng et al. [22] performed a comprehensive metabolomic analysis of coronary heart disease phlegm pattern and qi deficiency pattern and found that the endogenous metabolite serine, which distinguished the two patterns, was characterized mainly by abnormal amino acid metabolism. Su et al. [23] investigated the differential metabolites of patients with type 2 diabetes mellitus having qi and yin deficiency. Qi and yin deficiency pattern was related to protein and glucose metabolism disorders, insulin resistance, and intestinal flora disorder compared with non-qi and yin deficiency pattern. In addition, Sun et al. explored the changes in metabolites in patients with hepatitis B cirrhosis (31 cases) having four different patterns (spleen deficiency with dampness encumbrance pattern (SDDE), GSYX, GDSR, and blood stasis pattern (BS)) before and after using fuzhenghuayu tablet (FZHY) (24 weeks). They dynamically observed the therapeutic effect of FZHY on four different patterns. After treatment for 12 and 24 weeks, the metabolites of GSYX and SDDE were found to be significantly reversed, but not to GDSR and BS. This provided preliminary evidence for “fang zheng xiang ying” [24].

Because of the complexity of internal metabolites and the pattern of TCM, attempts were made to minimize the interference of accompanied symptoms and seek the material basis that could truly reflect the nature of pattern. This study used hepatitis B cirrhosis, a disease that could be treated using TCM, and two typical patterns of deficiency (GSYX pattern) and excess (GDSR pattern) for metabolomic analysis. Healthy persons were used as the control and then LP as the other control to eliminate the information not related to the pattern, so as to find out the specific substances that could reflect the nature of the disease and the pattern of TCM and hence identify the material basis of patterns of deficiency and excess in hepatitis B cirrhosis.

The results showed eight specific metabolites, including citrulline, urea, myristic acid, palmitic acid, palmitoleic acid, linoleic acid, glycolic acid, and petroselinic acid, in the GDSR pattern, removing information on metabolites from the healthy and disease groups (LP). These specific small-molecule compounds may be the intrinsic material basis of GDSR pattern. The metabolic pathway was linoleic acid metabolism, which was related to the biosynthesis of fatty acids. The synthesis of fatty acids uses Coenzyme A (CoA) as a carbon source and nicotinamide adenine dinucleotide phosphate (NADPH, reduced coenzyme II) as a hydrogen donor. They are synthesized in the cytosol of liver, brain, and fat. The liver is the most important site for the synthesis, and ATP provides the needed energy. The metabolites involved in fatty acid biosynthesis in this study included myristic acid, palmitoleic acid, and palmitic acid. All these are free fatty acids and important intermediate products of lipid metabolism, which are hydrolyzed by triglycerides in subcutaneous and visceral organs and then ingested and oxidized by the liver to supply energy. Palmitoleic acid is a monounsaturated fatty acid. Myristic acid and palmitic acid are saturated fatty acids, which can activate the NK-*κ*B signaling pathway in the cell by activating Toll-like receptors [25]. The NK-*κ*B signaling pathway is involved in a variety of physiological and pathological activities such as inflammation and cell survival [26]. Recent studies have shown that lipid metabolism disorders are closely related to inflammation [27], and inflammation is an important material basis for the damp-heat pattern [28]. A clinical investigation was conducted on the pattern characteristics and biological indicators of 223 and 440 patients with posthepatitis B cirrhosis. The results showed that AST and ALT were involved in hepatocyte inflammation in patients with GDSR (SRNY) pattern. The values of GGT, TBil, and DBil were significantly high, suggesting that hepatic inflammation in posthepatitis (hepatitis B) cirrhosis is the pathological basis of damp-heat pattern [29, 30]. This provided the clinical basis of the theory that inflammation is a material basis for the damp-heat pattern.

In this study, the contents of myristic acid, palmitoleic acid, and palmitic acid decreased in the GDSR group, referring to the fatty acid metabolism disorder, compared with the LP group. The metabolomic analysis was performed on serum samples of patients with CHB, NAFLD, and CG. Five common substances, including inosine and uridine, were involved in fatty acid metabolism disorders [15]. In addition, according to the theory of correspondence of the prescription and the pattern, several classical prescriptions were used to treat dimethyl nitrosamine- (DMN-) induced fibrotic model rats. Yinchenhaotang, a decoction that clears heat and promotes diuresis, could effectively inhibit liver fibrosis in DMN model rats. Also, differential genes were involved in the regulation of fatty acid metabolism, indicating that rats with DMN-induced liver fibrosis had characteristics of SR pattern, and an abnormal fatty acid metabolism might be one of the biological basis of this pattern [31], consistent with the results of the present study. Xu et al. [32] and Zhao et al. [33] also found abnormalities in fatty acid metabolism and lipid metabolism disorders in patients with type 2 diabetes, mandatory spondylitis, and gouty arthritis.

Similarly, eight specific metabolites, including L-threonine, pyroglutamic acid, L-arabitol, 1,5-anhydrosorbitol, glyceric acid, L-pipecolic acid, glutaric acid, and alpha-tocopherol, were found in GSYX, and the metabolic pathways were those of glycine, serine, and threonine. Glycine is a nonessential amino acid that protects multiple organs (liver, kidney, and so on) from ischemia and reperfusion [34]. Huang et al. [35] found that glycine injection through the tail vein of rats with severe pancreatitis exerted protective effects on hepatocytes. Recent studies showed immunomodulatory [36] and anti-inflammatory effects of glycine [37]. Glycine produces heme with nephrotoxicity, and its abnormal metabolic pathways may cause abnormal immune regulation and kidney damage. Previous studies showed that glycine inhibited liver fibrosis [38] and prevented alcoholic liver disease [39]. When liver tissue is damaged to a certain extent, glycine is consumed in large quantities for protecting against damage, and the content is significantly reduced. Therefore, the decrease in the glycine content in patients with GSYX suggests severe liver tissue damage and liver fibrosis. Additionally, glycine is an important component of glutathione in the metabolism of threonine and serine and has an antioxidant effect. A previous study showed the abnormal oxidative metabolism of GSYX in hepatitis B cirrhosis [40].

Threonine is an essential amino acid in the human body, with four isomers, and only the L-type has an effect on the body. It is the only amino acid that can transform into other substances without deamination and transamination. L-threonine binding to oligosaccharide chains has a protective effect on cell membranes and can promote phospholipid synthesis and fatty acid beta-oxidation. It has been used to treat fatty liver [41]. Glyceric acid is formed by the oxidation of glycerol, which is phosphorylated to form glycerate-3-phosphate, an intermediate product of serine degradation, and can further metabolize sugar or participate in glycolysis. The decrease in content reflects abnormal amino acid metabolism and carbohydrate metabolism.

GSYX of hepatitis B cirrhosis is more common in the later stages of the disease along with longer duration and weakness. A previous study confirmed that GSYX was reflected mainly in the decrease in the number and decline in the function of liver parenchyma cells and damage of the hepatic sinus wall [42, 43]. Clinical studies also found that decreased liver synthesis and liver parenchymal damage were the pathological basis of GXYX of hepatitis B cirrhosis [28, 29]. The level of amino acids in the blood decreased with the increase in protein consumption by the body and less ability of the liver to synthesize proteins. Wei et al. [44] found the common metabolites as glycine, tryptophan, and alanine in the metabolomic analysis of patients having GSYX after colorectal cancer and liver cancer surgery. Lu et al. [45] performed serum metabolomic analysis on patients with chronic kidney failure, chronic nephritis, chronic urinary tract infection, and diabetic nephropathy having GSYX. They found metabolic abnormalities of amino acids in the four diseases with GSYX. Song et al. conducted a metabolomic analysis on patients with cirrhosis having GSYX by TCM pattern differentiation and found abnormal amino acid metabolism in GSYX [38]. These results were all consistent with the finding of the present study in terms of amino acid metabolic pathway.

Six common substances, including D-2-hydroxyglutaric acid, 2-hydroxybutyric acid, L-phenylalanine, L-arabinose, L-serine, and quinic acid, were found by further comparing the two typical patterns. These substances, reflecting the common characteristics of the two patterns, are involved in the common metabolic pathways of glycine, serine, and threonine metabolism and phenylalanine metabolism, mainly manifesting as impairment of amino acid metabolic network pathway. Another study found common metabolites associated with amino acid metabolic pathways in patients with hepatitis B cirrhosis having GSYX [46].

In summary, either the GDSR pattern or the GSYX pattern has imbalances in homeostasis, and the metabolites and their characteristics are the results of internal environmental imbalances. Six common substances were found because the two patterns belonged to the same disease and had the same disease location and common symptoms in the clinic (dry mouth, fatigue, yellow urine, multiple dreams, irritability, and red tongue). The performance was also different after stimulation by the external environment because of the different internal environment of different patterns in the body. Hence, eight specific substances found in each of the two patterns represented the difference between the two groups of patterns. The present study only referred to typical patterns. Hence, further studies are needed to address the issue of concurrent patterns. Also, the sample size needs to be expanded to perform target validation and hence increase the reliability of the results. Later drug interventions should be carried out to provide a more reliable basis for the accurate identification of the typical patterns of hepatitis B cirrhosis.

## 5. Conclusions

The 22 differential metabolites obtained in the LP group in this study may serve as potential markers for hepatitis B cirrhosis. Eight potential biomarkers, including citrulline, urea, myristic acid, palmitic acid, palmitoleic acid, linoleic acid, glycolic acid, and petroselinic acid, were found in the GDSR pattern removing disease factors and common substances, and they all were involved in abnormalities of the linoleic acid metabolic pathway. Another eight potential biomarkers, including L-threonine, pyroglutamic acid, L-arabitol, 1,5-anhydrosorbitol, glyceric acid, L-pipecolic acid, glutaric acid, and alpha-tocopherol, were found in the GSYX pattern, and they all were involved in abnormalities of glycine, serine, and threonine metabolic pathways. These small-molecular compounds may serve as potential markers for distinguishing between GDSR and GSYX patterns. Six common substances, including 2-hydroxybutyric acid, L-serine, D-2-hydroxyglutaric acid, L-phenylalanine, L-arabinose, and quinic acid, were found in GDSR and GSYX patterns. They all were involved in the common metabolic pathways of glycine, serine, threonine, and phenylalanine, manifesting the commonality of the metabolic level of the two patterns.

## Figures and Tables

**Table 1:** Distribution of demographic characteristics in patients with hepatitis B cirrhosis and healthy volunteers.

	HG	WZ	GDSR	GSYX	X2/F
Gender (M/F)	40/20	24/6	30/10	26/15	3.08
Age (year)	34.05 ± 8.20	46.37 ± 10.21*∗*	46.98 ± 10.20*∗*	51.32 ± 9.16*∗*▲&	33.20
BMI (kg/m2)	22.04 (21.63, 23.01)	22.86 (22.11, 23.91)	23.67 (22.82, 25.68)	22.43 (22.00, 23.92)	5.07

*∗P* < 0.05, compared with the normal group; ▲*P* <0.05, compared with the LP group; & *P* <0.05, compared with the GDSR group.

**Table 2:** Physical and chemical examination results between different groups [*M* (*Q*_*1*_, *Q*_*3*_)].

Index	Group			
	HG	LP	GDSR	GSYX
TBil (*μ*mol/L)	11.38 (10.08, 14.12)	20.50 (11.24, 27.18)	28.05*∗*▲ (21.05, 49.40)	25.30*∗*& (18.87, 38.65)
DBil (*μ*mol/L)	2.22 (1.80, 2.49)	3.92 (2.65, 6.7)	8.58*∗*▲ (4.60, 19.77)	5.56& (4.35, 11.49)
ALT (IU/L)	18.00 (12.00, 25)	25.00 (16.50, 29.50)	30.95 (19.75, 43.25)	27 (19.50, 43.25)
AST (U/L)	20.00 (17.00, 23.00)	26.00 (21.25, 33.25)	40.50*∗*▲ (29.00, 56.25)	37.00 (26.00, 58.50)
ALP (U/L)	77.00 (66.00, 92.00)	77.00 (56.50, 91.50)	100.00*∗*▲ (77.00, 148.50)	91.00*∗* (71.50, 111.50)
GGT (U/L)	18.28 (14.04, 27.01)	31.25 (21.50, 54.97)	40.00*∗* (28.00, 52.67)	34.62*∗* (21.35, 65.79)
TBA (*μ*mol/L)	3.35 (2.10, 6.74)	12.00 (6.75, 19.88)	44.65*∗*▲ (14.56, 104.61)	43.19*∗*▲ (10.01, 82.21)
ALB (g/L)	47.31 (45.36, 48.89)	44.95*∗* (39.98, 47.76)	36.093*∗*▲ (30.97, 40.70)	35.34*∗*▲ (26.83, 42.65)

*∗P* < 0.05, compared with the normal group; ▲*P* < 0.05, compared with the LP group; &*P* < 0.05, compared with the GDSR group.

**Table 3:** Different metabolites between LP and HG.

No.	Differential metabolites	VIP	P	FC	Tendency	Class
1	Beta-alanine	2.6	≤0.001	0.32	↓	Amino acid
2	Creatinine	1.8	≤0.001	0.68	↓	Amino acid
3	L-Asparagine	1.6	≤0.001	1.33	↑	Amino acid
4	L-Tyrosine	1.7	0.001	1.44	↑	Amino acid
5	D-Threitol	1.8	≤0.001	1.70	↑	Carbohydrate
6	Rhamnose	2.0	≤0.001	1.72	↑	Carbohydrate
7	L-Sorbose	1.7	≤0.001	0.53	↓	Carbohydrate
8	D-Fructose	1.7	≤0.001	0.53	↓	Carbohydrate
9	Gluconolactone	1.8	≤0.001	1.86	↑	Carbohydrate
10	Sucrose	1.6	≤0.001	2.01	↑	Carbohydrate
11	D-Maltose	1.7	≤0.001	79.59	↑	Carbohydrate
12	Pelargonic acid	2.0	≤0.001	1.90	↑	Fatty acid
13	Tetracosanoic acid	1.8	≤0.001	1.32	↑	Fatty acid
14	Normetanephrine	1.7	≤0.001	0.72	↓	Hormone
15	Tryptamine	1.6	≤0.001	0.62	↓	Indoles
16	Uracil	1.6	0.001	1.97	↑	Nucleotide
17	Inosine	2.0	≤0.001	5.15	↑	Nucleotide
18	Malic acid	1.6	0.002	1.78	↑	Organic acids
19	Oxoglutaric acid	1.8	≤0.001	3.22	↑	Organic acids
20	Taurine	1.8	≤0.001	0.45	↓	Organic acids
21	Citric acid	1.8	≤0.001	2.51	↑	Organic acids
22	Isocitric acid	2.1	≤0.001	2.76	↑	Organic acids

FC, fold change; VIP, variable importance in projection. ↑, the content of this metabolite was higher in the group of latent pattern (LP) than in the healthy group; ↓, the content of this metabolite was lower.

**Table 4:** Different metabolites between LP and GDSR.

No.	Differential metabolites	VIP	P	FC	Tendency	Class
1	2-Hydroxybutyric acid	1.72	0.004	0.71	↓	Amino acid
2	Urea	1.79	0.027	0.46	↓	Amino acid
3	L-Serine	1.67	0.002	0.78	↓	Amino acid
4	D-2-Hydroxyglutaric acid	1.75	0.002	0.70	↓	Amino acid
5	L-Phenylalanine	1.93	≤0.001	0.58	↓	Amino acid
6	L-Asparagine	1.50	0.007	0.82	↓	Amino acid
7	Citrulline	1.5	0.026	0.79	↓	Amino acid
8	L-Tyrosine	2.12	≤0.001	0.68	↓	Amino acid
9	L-Arabinose	1.84	0.002	0.79	↓	Carbohydrate
10	L-Sorbose	1.73	0.001	0.54	↓	Carbohydrate
11	D-Fructose	1.73	0.001	0.54	↓	Carbohydrate
12	Pelargonic acid	2.24	≤0.001	1.71	↑	Fatty acid
13	Myristic acid	1.86	0.001	0.49	↓	Fatty acid
14	Palmitoleic acid	1.78	0.001	0.36	↓	Fatty acid
15	Palmitic acid	1.77	≤0.001	0.59	↓	Fatty acid
16	Linoleic acid	1.65	0.002	0.75	↓	Fatty acid
17	Tryptamine	2.10	0.001	0.64	↓	Indoles
18	Glycolic acid	1.72	0.001	0.68	↓	Organic acid
19	Quinic acid	1.63	0.004	0.77	↓	Organic acid
20	Petroselinic acid	1.94	0.001	0.50	↓	Organic acid

**Table 5:** Different metabolites between LP and GSYX.

No.	Differential metabolites	VIP	P	FC	Tendency	Class
1	2-Hydroxybutyric acid	1.88	≤0.001	0.56	↓	Amino acid
2	L-Serine	1.66	0.011	0.83	↓	Amino acid
3	L-Threonine	1.57	0.017	0.77	↓	Amino acid
4	Pyroglutamic acid	1.71	0.014	0.77	↓	Amino acid
5	D-2-Hydroxyglutaric acid	1.91	0.001	0.72	↓	Amino acid
6	L-Phenylalanine	2.57	≤0.001	0.59	↓	Amino acid
7	L-Asparagine	1.68	0.008	0.81	↓	Amino acid
8	L-Tyrosine	1.54	0.006	0.67	↓	Amino acid
9	L-Arabinose	2.37	≤0.001	0.79	↓	Carbohydrate
10	L-Arabitol	2.00	0.001	0.69	↓	Carbohydrate
11	1,5-Anhydrosorbitol	1.73	0.005	1.98	↑	Carbohydrate
12	D-Fructose	2.72	≤0.001	2.02	↑	Carbohydrate
13	Pelargonic acid	1.78	≤0.001	0.62	↓	Fatty acid
14	Glyceric acid	1.85	0.015	0.65	↓	Organic acid
15	L-Pipecolic acid	1.54	0.003	0.75	↓	Organic acid
16	Glutaric acid	1.70	0.001	0.16	↓	Organic acid
17	Quinic acid	1.62	0.21	0.84	↓	Organic acid
18	Alpha-Tocopherol	1.88	≤0.001	0.56	↓	Vitamin

**Table 6:** Common and specific metabolites between GDSR and GSYX.

GDSR		Common metabolites	GSYX	
Common with disease	Specific metabolites		Specific metabolites	Common with disease
L-Sorbose Tryptamine	Palmitoleic acid	D-2-Hydroxyglutaric acid	1,5-Anhydrosorbitol	No
	Myristic acid	L-Asparagine	L-Arabitol	
	Citrulline	2-Hydroxybutyric acid	L-Threonine	
	Palmitic acid	L-phenylalanine	L-Pipecolic acid	
	Petroselinic acid	L-arabinose	Pyroglutamic acid	
	Linoleic acid	L-Serine	Alpha-tocopherol	
	Glycolic acid	L-Tyrosine	Glyceric acid	
	Urea	D-Fructose	Glutaric acid	
		Quinic acid		
		Pelargonic acid		

## Data Availability

The data were detected in March 2017. The data used to support the findings of this study are available from the corresponding author upon request.

## Supplementary Materials

Supplementary Materials In order to analyze metabolites in serum, samples needed to be pretreated. Detailed procedure was provided in the supplementary file.
