# Supplementary material for: Classification of Gan Dan Shi Re Pattern and Gan Shen Yin Xu Pattern in Patients with Hepatitis B Cirrhosis Using Metabonomics
Source: Evid Based Complement Alternat Med. 2018 Nov 21;2018:2697468. doi: 10.1155/2018/2697468 (PMC6280296; doi:10.1155/2018/2697468)
Supplement: Supplementary Materials — In order to analyze metabolites in serum, samples needed to be pretreated. Detailed procedure was provided in the supplementary file. [file 2697468.f1.doc]

**Sample Pretreatment**

Each 50 μl aliquot serum sample was mixed with 10-μl of internal standard, and then another 175-μl pre-chilled mixture of methanol: chloroform (v/v=3:1) was added for protein precipitation and vortexed for 30s. After stored for 20 min at -20 °C, the mixture was centrifugated at 14,000 g and 4 °C for 20 minutes. 200 ul of supernatant was accurately transferred to a 2ml sample vial and evaporated in a centrifugal concentrator for 5 minutes to remove chloroform, and then transferred to a low temperature freeze dryer for lyophilization. The completely dried samples were dissolved in 50 ul methoxyamine solution (20mg/ml in pyridine), incubated at 30°C for 2 hours. The resulting products were further silylated with 50 ul MSTFA (containing 1% TMCS) splked with FAMEs as retention indices at 37.5°C for 1 hour. A 1-μl aliquot of derivatized mixture was analyzed by GC-TOFMS.
